# Development of a Vacuum Electrospray Droplet Ion Gun for Secondary Ion Mass Spectrometry

**DOI:** 10.5702/massspectrometry.A0069

**Published:** 2018-07-31

**Authors:** Satoshi Ninomiya, Yuji Sakai, Lee Chuin Chen, Kenzo Hiraoka

**Affiliations:** 1 Interdisciplinary Graduate School, University of Yamanashi, 4–3–11 Takeda, Kofu, Yamanashi 400–8511, Japan; 2 Clean Energy Research Center, University of Yamanashi, 4–3–11 Takeda, Kofu, Yamanashi 400–8511, Japan

**Keywords:** vacuum electrospray, massive cluster ion beam, secondary ion mass spectrometry

## Abstract

Atmospheric pressure electrospray had been used in previous studies to generate massive water droplet ion beams, and the beams successfully achieved efficient desorption/ionization of biomolecules, low damage etching of polymers and nonselective etching of metal oxides. However, this droplet ion beam was not practical as a primary ion beam for surface analysis instruments because it required differential pumping and lacked adequate beam current and density. To improve the beam performance, we have proposed to use vacuum electrospray of aqueous solutions as a beam source, and developed a technique for producing a stable electrospray of aqueous solution in vacuum. We also designed a prototype of a vacuum electrospray droplet ion gun, and measured the beam properties. Finally, the applicability of this ion gun in secondary ion mass spectrometry is discussed.

## INTRODUCTION

Ion beams are indispensable for sample etching and ionization of molecules in the surface analysis methods such as secondary ion mass spectrometry (SIMS) and X-ray photoelectron spectroscopy (XPS). The ion beams are composed of single atoms or clusters of atoms and molecules, and in principle, all atoms, molecules, and clusters can be used as ion beams. In fact, due to the development of various ion beams, the performance of surface analysis has been greatly improved. Compared to atomic ion beams, cluster ion beams have advantages that can etch organic materials with low damage and efficiently ionize biomolecules. Since the early 2000s, cluster ion guns capable of generating C_60_, Au cluster and Bi cluster beams have been put into practical use one after another for the purpose of application in surface analysis.^1–6)^ C_60_ ion beams have been frequently used for the etching of organic materials in SIMS and XPS depth profile analysis.^6,7)^ In order to achieve precise depth profiling, the optimization of experimental conditions such as incident energy, incident angle, sample rotation speed and sample cooling temperature was often required.^8–11)^ Au and Bi clusters produced by liquid metal ion sources (LMISs) have been used as ionization probes in SIMS, because they can increase the ionization efficiency of biomolecules and are suitable for imaging analysis.^4–6)^ However, even with these cluster beams, it is difficult to perform imaging analysis with a high spatial resolution (≤1 μm).

Massive cluster ion beams have been also studied to further increase ionization efficiencies, expand the mass range and achieve damage-less etching. In 1991, Mahoney and coworkers generated a massive cluster beam by using electrohydrodynamic emission of glycerol solutions.^12)^ It was reported that the charged glycerol droplet could achieve soft desorption/ionization of peptides and proteins up to 17,000 u,^13)^ and this technique was called the massive cluster impact (MCI) method. Despite its promising capabilities, it was not practically used as an ionization source for mass spectrometry, because electrospray ionization (ESI)^14,15)^ and matrix-assisted laser desorption/ionization (MALDI)^16,17)^ methods had been developed in the 1990s. Recently, however, Williams and coworkers suggested a potential application for microscopic molecular imaging by using the MCI method combined with a microscope-mode time-of-flight mass spectrometer (TOFMS).^18,19)^ They obtained a spatial resolution of ∼3 μm for protonated intact molecule images of a bradykinin feature in 1 min. Fujiwara and coworkers reported that the massive cluster ion beam generated by the vacuum electrospray of a protic ionic liquid was useful in increasing protonated molecular species.^20)^ Massive gold cluster ions have been studied using LMIS techniques by several research groups.^21)^ Precursor peptide molecular ion yield enhancements of 1,000 were measured by comparing SIMS spectra obtained using Au^+^ and Au^4+^_400_ primary ions,^22)^ and very high secondary ion yields (>1) were also obtained for various biomolecules in experiments run in the event-by-event bombardment-detection mode.^23)^ The gas cluster ion beam (GCIB) technique, originally developed for surface modification by Yamada and coworkers,^24)^ is very successful in the damage-less etching of organic materials. Various kinds of gas such as Ar, O_2_, and SF_6_ can be used as a source gas for GCIB. By using Ar-GCIB for etching in SIMS and XPS depth analysis, accurate depth profiles can be obtained relatively easily for any organic samples.^25–27)^ At present, most surface analysis manufactures sell SIMS and XPS instruments equipped with compact Ar-GCIB guns. Of course, GCIB has been also studied as a SIMS probe, and it was already shown that it could suppress fragment ions and could ionize large biomolecules such as proteins.^28)^ Furthermore, the ionization efficiencies when using water vapor, CH_4_ and CO_2_ as the source (or doping) gas of GCIB have been investigated to improve the sensitivity in SIMS.^29–31)^ The H_2_O-GCIB enhanced the secondary ion yields by a factor of 10 or more over Ar-GCIB for several biomolecular samples.^29)^

The electrospray droplet impact (EDI) method was developed by Hiraoka and coworkers as a new massive cluster ion beam, in which the water droplet ion beams were produced from an electrospray of an aqueous solution at atmospheric pressure.^32)^ The droplet ion beams were introduced through an orifice of 400 μm diameter into the first vacuum chamber, transported by a quadrupole ion guide, and accelerated up to 10 kV after exiting the ion guide. The beam current on the target was typically 1 nA, and the beam diameter was around 2 mm without the object lens, and 0.2 mm with the object lens. This atmospheric pressure EDI (A-EDI) beam gun was connected to an orthogonal acceleration TOFMS and a typical XPS. The A-EDI beams have successfully achieved efficient desorption/ionization of biomolecules,^32,33)^ low damage etching of polymers^34,35)^ and nonselective etching of metal oxides.^36,37)^ However, the A-EDI gun was not practical as a primary ion beam gun for surface analysis instruments because it required differential pumping and lacked adequate beam current and density. The low beam current and density of the A-EDI gun is attributed to the use of an atmospheric electrospray as the beam source because the droplet ion beams are apparently lost by dispersion in air and in the differential pumping chamber before reaching the sample chamber at high vacuum. From the above-mentioned point, to improve the water droplet ion beam current and density, we have proposed to use vacuum electrospray of aqueous solutions as a beam source.^38)^ We developed a technique for producing a stable electrospray of aqueous solution in vacuum, designed the prototype of a vacuum electrospray droplet ion (V-EDI) gun,^39,40)^ and explored the applicability of this V-EDI gun in SIMS.^41,42)^

## VACUUM ELECTROSPRAY

In general, electrospray of a volatile liquid is carried out at atmospheric pressure. When a high voltage is applied to a metal capillary filled with a liquid, an excess charge is accumulated in the liquid and the force toward the outside due to the Coulomb repulsion and inward force due to the surface tension of the liquid are balanced, resulting in the formation of a conical shape liquid (Taylor cone)^43)^ at the tip of the capillary. The point at which this occurs is known as the Rayleigh limit.^44)^ Furthermore, when the Coulomb repulsion exceeds the surface tension of the liquid tip, charged droplets are sprayed from the tip of the Taylor cone.^45)^ This phenomenon is called “electrospray.” In ESI, the charged droplets shrink due to solvent evaporation and break up into smaller droplets as due to the presence of excess charges (Rayleigh fission).^45)^ By repeating desolvation and splitting of the charged droplets, isolated molecular ions are finally formed. The ESI technique, which was first reported by Fenn and coworkers is one of the most important techniques for ionizing organic molecules and has been widely used for biological mass spectrometry.^14,15)^

### Verification experiment of vacuum electrospray

**Figure 1:**
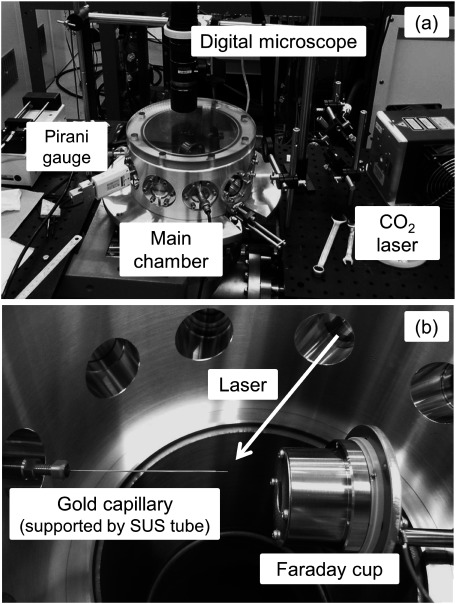
Fig. 1. The first experimental setup for vacuum electrospray. Photographs of the configuration (a) and the inside of the main chamber (b). A gold capillary supported by stainless steel tube and a Faraday cup was installed in the chamber. (Reproduced from Ninomiya *et al.* (Ref. 40), copyright 2017, with permission from The Vacuum Society of Japan.)

We have proposed to use vacuum electrospray of aqueous solutions as a source of a novel massive cluster ion beam gun.^38)^ However, vacuum electrospray of aqueous solutions is very difficult because of following two major problems: (i) the freezing of the aqueous solutions introduced in the vacuum system by evaporative cooling and (ii) the occurrence of the electric discharge under low vacuum conditions (Paschen’s law).^46)^ To avoid freezing of the aqueous solutions introduced in vacuum, the tip of the capillary was irradiated by laser, and to avoid the electric discharge, the vacuum chamber was maintained at high vacuum by relatively large vacuum pumps. Figure 1(a) shows the photograph of the first experimental setup for vacuum electrospray. The main chamber was evacuated with an 800 L/s turbo molecular pump (TMP, STP-H803C, Edwards, Crawley, UK), a 4,700 L/min mechanical booster pump (MBP, PMB-003C, ULVAC, Chigasaki, Japan), and a 460 L/min rotary pump (RP, E2M28, Edwards) connected in series. The chamber pressure was monitored by a Pirani gauge (M-350PG-SD/N25, ANELVA, Kawasaki, Japan). In our experimental setup, a pressure lower than 0.1 Pa was needed to apply sufficient high voltage. By using these three pumps, the working pressure was maintained lower than 0.05 Pa even at high liquid flow-rate conditions up to 10 μL/min.^38)^ To avoid freezing and maintain the fluidity of the volatile liquids introduced in vacuum, infrared (IR) CO_2_ laser (center wavelength λ=10.6 μm, FSV20KFB, Synrad, Mukilteo, WA) was used, because water absorbs this wavelength of light very well (absorption coefficient of water: 832 cm^−1^).^47)^ The laser was reflected two times by the gold-coated mirrors and focused by a condenser lens outside the main chamber. The laser was finally transmitted into the main chamber through a ZnSe window, and the tip of the electrospray emitter was irradiated by the IR laser in vacuum. The laser path was adjusted before evacuating the chamber such that the laser beam was focused at the tip of the electrospray emitter. An electrospray emitter and a Faraday cup (aperture size 10 mmφ, length 20 mm) were installed in the main chamber as shown in Fig. 1(b). The electrospray emitter used in this study was a gold capillary (inner diameter (i.d.) 200 μm) because Au efficiently reflects IR light. The Faraday cup was around 10 mm away from the tip of the capillary. Liquids were supplied to the electrospray emitter by a syringe pump (PHD ULTRA, HARVARD Apparatus, Holliston, MA).

**Figure 2:**
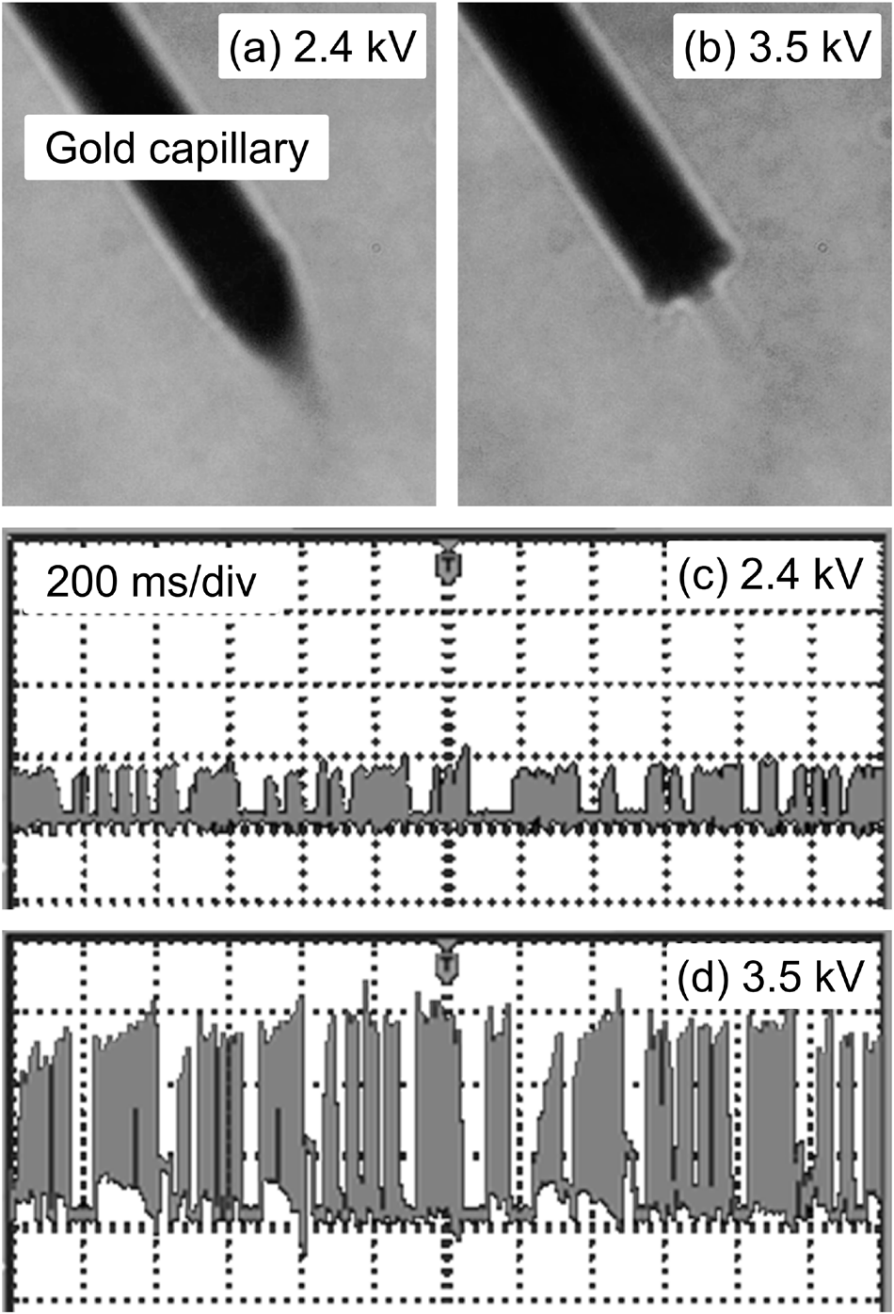
Fig. 2. Snapshots of the Taylor cone formed at the tip of the gold capillary (i.d. 200 μm) at 2.4 (a) and 3.5 kV (b) observed with the optical digital microscope system. Electrospray current intensities (1 V/division) as a function of time (200 ms/division) recorded by an oscilloscope at 2.4 (c) and 3.5 kV (d). A 0.01 M TFA water/methanol (1/1) mixed solution was supplied at a flow rate of 10 μL/min.

The tip of the electrospray emitter was directly observed with an optical digital microscope system (VH-5500 coupled with VH-Z50L lens, Keyence, Osaka, Japan) with a maximum magnification of 500 and a working distance of 85 mm. The microscope system allows accurate identification of the Taylor cone even under vacuum. As the first step of vacuum electrospray, a 0.01 M trifluoroacetic acid (TFA) water/methanol (1/1) solution (flow rate: 10 μL/min) was used, because the freezing point of methanol is much lower than water. A high-voltage was applied to the capillary through the stainless steel shaft. Before laser irradiation, gel-like matter was formed at the tip of the capillary. Once the gel-like matter was melted by laser irradiation, electrospray was continuously produced without laser irradiation. Figures 2(a) and 2(b) show the snapshots of the Taylor cone formed at the tip of the capillary at 2.4 kV and 3.5 kV, respectively. It was clearly confirmed that Taylor cone was formed at the tip of the capillary. Although laser irradiation was used only for melting the gel-like matter at the beginning stage, vacuum electrospray of the mixed solution could be achieved. However, the shapes of the Taylor cone were very unstable even under the same flow rate and electrospray voltage. The currents measured with a picoammeter (6485, Keithley, Cleveland, OH) at 2.4 and 3.5 kV were 90–120 nA and 250–500 nA, respectively. When the electrospray current at the Faraday cup is amplified with a low-noise current amplifier (SR570, Stanford Research Systems, Sunnyvale, CA) and monitored with an oscilloscope (DSO1024A, Agilent Technologies, Santa Clara, CA), the time-resolved changes in current at fixed voltage can be visualized. Figures 2(c) and 2(d) show the electrospray currents recorded in real time by the oscilloscope at 2.4 and 3.5 kV, respectively. The sensitivity of the current amplifier was 2 μA/V, and the time scale of the oscilloscope was 200 ms/division. It was found that the currents of vacuum electrospray frequently fluctuated. Moreover, in the absence of methanol, the electrospray current was more unstable than the above results. Nevertheless, it was demonstrated that vacuum electrospray of the aqueous solutions could be realized by maintaining appropriate vacuum conditions to prevent the electric discharge and by heating the tip of the capillary with laser to prevent freezing of the aqueous solutions.

### Vacuum electrospray as a beam source

**Figure 3:**
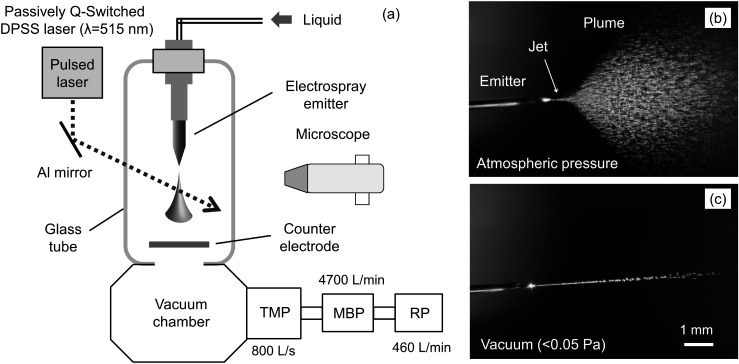
Fig. 3. Schematic of an optical microscopy setup (a) and the images of atmospheric-pressure electrospray (b) and vacuum electrospray (c) acquired with pulsed laser illumination. (Reproduced from Ninomiya *et al.* (Ref. 40), copyright 2017, with permission from The Vacuum Society of Japan.)

In general, electrospray of aqueous solutions is carried out at atmospheric pressure, but in the previous section, it was confirmed that electrospray of aqueous solutions could be achieved even under vacuum. In this section, the properties of vacuum electrospray as an ion beam source are evaluated. Figure 3(a) shows an experimental setup for optical microscopy to observe the difference between atmospheric pressure and vacuum electrospray.^48)^ A commercially available metal-coated silica emitter (i.d. 30 μm, New Objective, Woburn, MA) was installed in a glass tube (NW40) connected to the main chamber, and the main chamber was evacuated with an 800 L/s TMP, a 4,700 L/min MBP, and a 460 L/min RP connected in series. The tip of the emitter was illuminated using a passively Q-Switched DPSS laser (λ=515 nm, 1.0 mJ maximum energy, Halo GN, InnoLight GmbH, Hannover, Germany) with a repetition rate of 10 Hz and with a pulse width of less than 10 ns. The electrospray generated from the emitter tip was visualized with this laser. The scattering light from the electrospray droplets was captured using a digital camera, and the camera shutter was controlled in such a way that only one laser shot was recorded in each camera shot. In this study, pure methanol was used as an electrospray solution, because the freezing point is much lower than that of pure water and electrospray could be relatively easily performed even in vacuum. In the case of methanol, it rarely froze at the tip of the emitter.

Figures 3(b) and 3(c) show the snapshots of electrospray produced at the tip of the emitter under atmospheric pressure and vacuum conditions, respectively. Each electrospray was clearly visualized with pulsed laser. Charged droplets exceeding Rayleigh limit are released as “jet” from the tip of a Taylor cone. For atmospheric pressure electrospray, the charged droplets shrink because of solvent evaporation and break up into smaller droplets when exceeding the Rayleigh limit again.^45)^ Very small charged droplets produced by repetition of the shrinkage and fission are called “plume,” and it is well known that the charged droplets are rapidly decelerated by collisions with molecules in the atmosphere and driven away from each other by Coulomb repulsion.^45)^ As clearly shown in the Fig. 3(b), the jet generated from the tip of the emitter started to diffuse after about 1 mm flight, and the cone-like plume was observed away from the tip of the emitter. On the other hand, the snapshot of vacuum electrospray was dramatically different from that of atmospheric pressure electrospray.^48)^ Under vacuum, the straight jet was formed at least with the length of 5 mm from the tip of the emitter, as shown in Fig. 3(c). This must be caused by the fact that the space charge field effect became negligible for the charged droplets that were accelerated by the high electric field in vacuum. The initial velocity of the charged droplets should be sustained because they rarely collide with residual gases in high vacuum. This result is the rationale for the development of vacuum electrospray that is suitable for focusing of the beam leading to the high beam density.

## VACUUM ELECTROSPRAY DROPLET ION GUN

### Prototype of vacuum electrospray droplet ion gun

**Figure 4:**
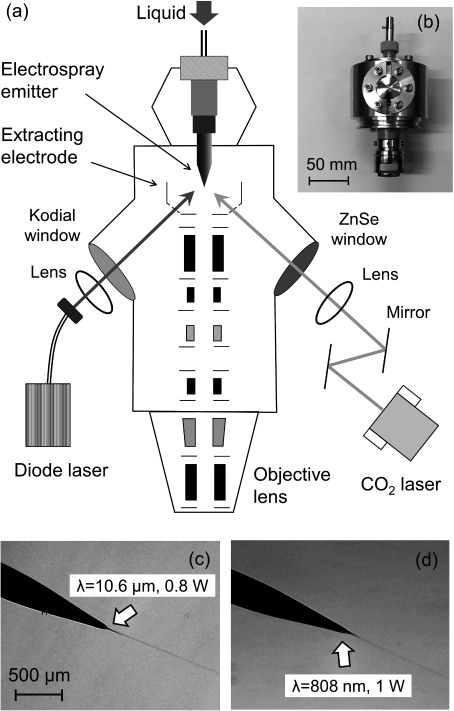
Fig. 4. Schematic of a prototype of the vacuum electrospray droplet ion gun (a), photograph of the source part (b), and the images of vacuum electrospray assisted by IR laser (c) and near-IR laser (d). (Reproduced from Ninomiya *et al.* (Ref. 40), copyright 2017, with permission from The Vacuum Society of Japan.)

This research aims at the development of a novel massive cluster ion gun that can be utilized in SIMS. Therefore, the practical performances, such as easy operation, long lifetime, stable beam current and small equipment size, are very important. Based on the above results, we have designed and fabricated a prototype of a vacuum electrospray droplet ion (V-EDI) gun.^39,40)^ Figure 4(a) shows the schematic view of the V-EDI gun that was designed for SIMS applications, with a total length of ∼600 mm, the similar size as a conventional liquid metal ion gun. The main part of the V-EDI gun was made of aluminum for weight and cost reduction. The main part of the V-EDI gun was evacuated using a 300 L/s TMP (HiPace 300, Pfeiffer, Asslar, Germany) and a 460 L/min RP. The photograph of Fig. 4(b) shows the source part of the V-EDI gun. The volume of the source part reduced to less than 1/4 of that of the A-EDI gun, and the weight also decreased to less than half (1.1 kg) of the previous one (2.4 kg). An electrospray emitter was installed in the source part of the V-EDI gun. To maintain appropriate vacuum conditions by this pumping system, it was necessary to reduce the liquid supply from the electrospray emitter. To decrease the flow rate of the liquid less than a few μL/min, several commercially available fine electrospray emitters were examined,^39)^ *i.e.*, metal-coated fused silica (i.d. 15, 30 μm, New Objective). Each electrospray emitter was joined to a stainless steel fitting, which was in turn connected to a stainless steel shaft embedded in a custom-made NW25 PEEK flange. The PEEK flange was designed to withstand a high voltage of 10 kV or higher. The distance between the tip of the electrospray emitter and the extracting electrode was adjustable to optimize the electrospray conditions. In this study, to prevent freezing of the liquid, the tip of the electrospray emitter was irradiated by a continuous wave (CW) IR CO_2_ laser (λ=10.6 μm) or a CW near-IR diode laser (λ=808 nm, 3 W maximum power, LFP-808W, Neoark, Tokyo, Japan) instrument. Each laser was introduced through a ZnSe or glass window, at an irradiation angle of 55° with respect to the direction of electrospray. The vacuum electrospray was directly observed with the digital microscope system.

In the previous study, an IR laser (λ=10.6 μm) was adopted to maintain the fluidity of the aqueous solutions because water absorbs this wavelength of light very well. When the laser intensity was carefully adjusted such that the tip of the metal-coated silica emitter would not melt, a stable vacuum electrospray of aqueous solutions could be achieved.^38)^ However, fine adjustment of the IR laser irradiation is too tedious under vacuum conditions, mainly because the IR laser light is not visible. In addition, the CW CO_2_ laser system is hard to miniaturize, and therefore it is unsuitable for practical use, which was focused on the capacity of the CW near-IR diode laser to be used for vacuum electrospray of aqueous solutions.^39)^ The irradiation position of the near-IR diode laser in the visible range can be observed with the digital microscope, and a compact diode laser system meets the miniaturization requirements of the V-EDI gun. The center wavelength of this diode laser is 808 nm, and the absorption coefficient in water at 808 nm is 4 orders of magnitude less than that at 10.6 μm.^47)^ That is, the aqueous solutions are assumed to be indirectly heated by heat transfer from the tip of the electrospray emitter. It should be confirmed whether the diode laser can be used for vacuum electrospray. The source liquid used in this study was a 0.01 M TFA aqueous solution (flow rate: 0.5 μL/min) along with the metal-coated silica emitter with 30 μm i.d. A solid matter was formed at the tip of the electrospray emitter before irradiating with the laser. In such a situation, electrospray did not occur even when a high voltage was applied to the emitter. After that, when each laser was irradiated to the tip of the emitter with an intensity of 1 W or less, a typical cone-jet mode electrospray was generated. Figures 4(c) and 4(d) show the microscope images of vacuum electrospray assisted by CO_2_ and diode laser irradiation, respectively. Very similar cone-jet mode of vacuum electrospray was observed with both laser irradiation. The effect of bias voltage on the electrospray current for the diode laser irradiation was in good agreement with that found for the CO_2_ laser irradiation. Each electrospray current was more than 500 nA, and this value was equivalent to that obtained with the gold capillary (i.d. 200 μm, flow rate: 10 μL/min). It was confirmed that high current could be obtained even when the liquid flow rate was much reduced.

### Beam properties

**Figure 5:**
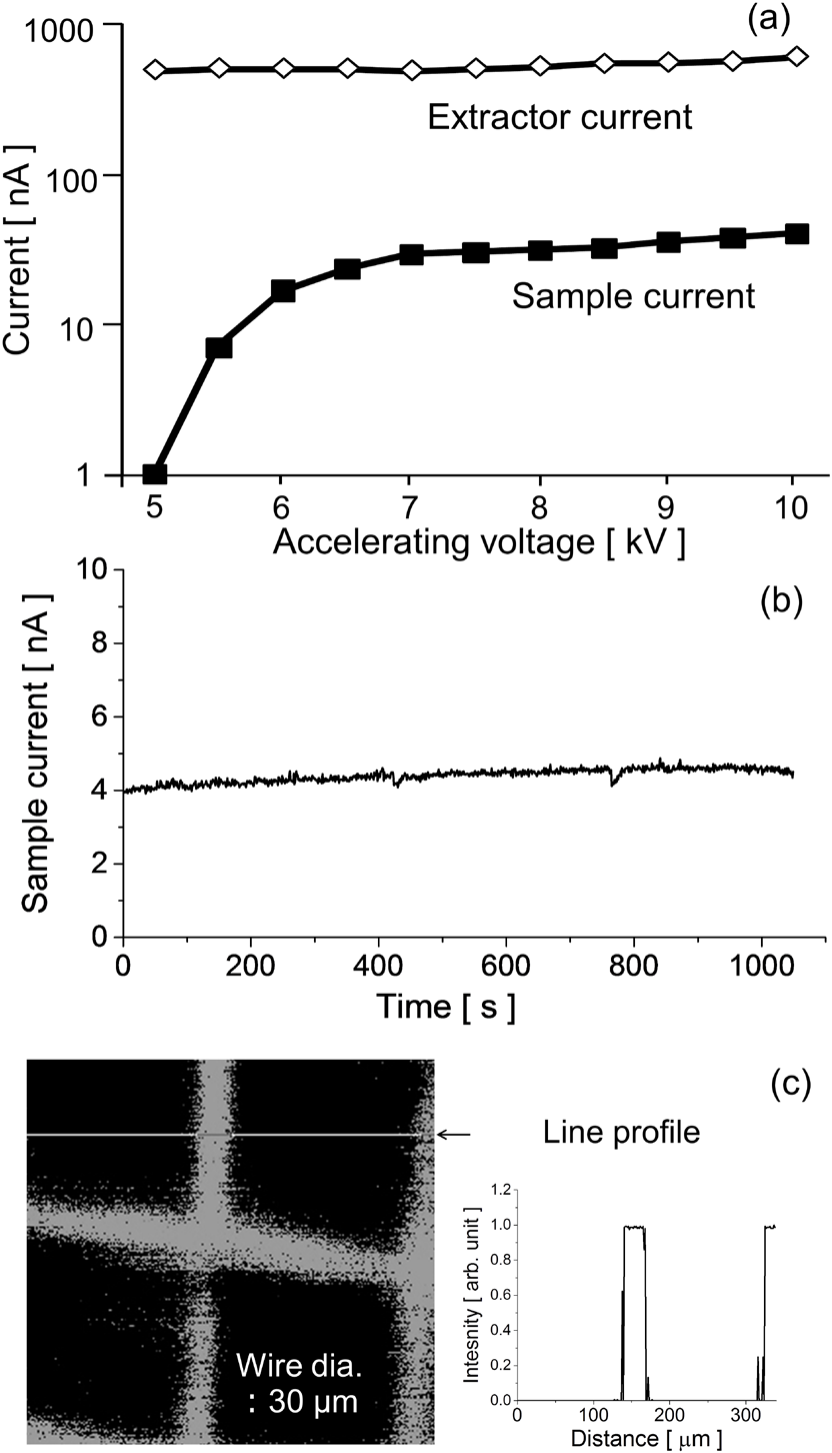
Fig. 5. The effects of accelerating voltage on the extractor current and sample current (a), sample current recorded for 1,000 s (b), and the secondary electron image and its line profile for the mesh sample irradiated by the V-EDI beam (c).

The beam currents on the extractor and sample holder were checked for the V-EDI gun when an electrospray voltage of 2.0 kV was applied to the emitter. The effects of accelerating voltage on the extractor and sample currents are shown in Fig. 5(a). The extractor current kept constant with increasing accelerating voltage, while the sample current increased with accelerating voltage and saturated at around 7.0 kV. More than 30 nA sample current was obtained for the V-EDI gun, and this current was at least 10 times higher than that for the A-EDI gun. The beam stability of the V-EDI gun was evaluated by monitoring the sample holder current. As the permanent liquid supply to the source is crucial for practical use, the stability of the beam current was checked by using a liquid chromatography pump (Micro-Flow pump MP710, GL Sciences, Tokyo, Japan). Figure 5(b) shows the sample currents as a function of time recorded by the picoammeter. Very stable sample currents within 10% variation were obtained for more than 1,000 s. Moreover, continuous vacuum electrospray was obtained using the silica emitter and the LC pump for more than 15 h. It is confirmed that the beam stability of the V-EDI gun is comparable to those of typical cluster ion guns.

The focusing properties of the V-EDI beams were also evaluated by ion beam induced secondary electron image. In this study, the beam was collimated by an aperture of 0.1 mm diameter and was finally focused by an objective lens with 15 mm working distance installed at the end of the gun (Fig. 4(a)). The focused V-EDI beam was raster-scanned on a stainless steel mesh (wire diameter 30 μm), and the emitted secondary electrons were detected with a scintillator-photomultiplier assembly installed near the sample holder. The V-EDI beam induced secondary electron image of the mesh sample and its line profile of the image are shown in Fig. 5(c). A clear image of the mesh sample was observed, and the sharp line profile of the image was also obtained. From the full width at half maximum of the profile, the beam diameter was assumed to be less than 10 μm. In addition, the density of the V-EDI beam was calculated to be more than 2 mA/cm^2^ (0.4 nA), and the beam density was at least two orders of magnitude higher than that of the A-EDI beam. By reducing the working distance of the objective lens to 10 mm, it might be possible to focus the V-EDI beam to less than 5 μm. Moreover, the vacuum electrospray started within 1 min after 1 day interval, and the reproducibility of the starting process was also confirmed. Based on these results, it is concluded that the vacuum electrospray technique has a promising future for high-performance massive cluster ion gun for SIMS.

## SECONDARY ION MASS SPECTROMETRY

**Figure 6:**
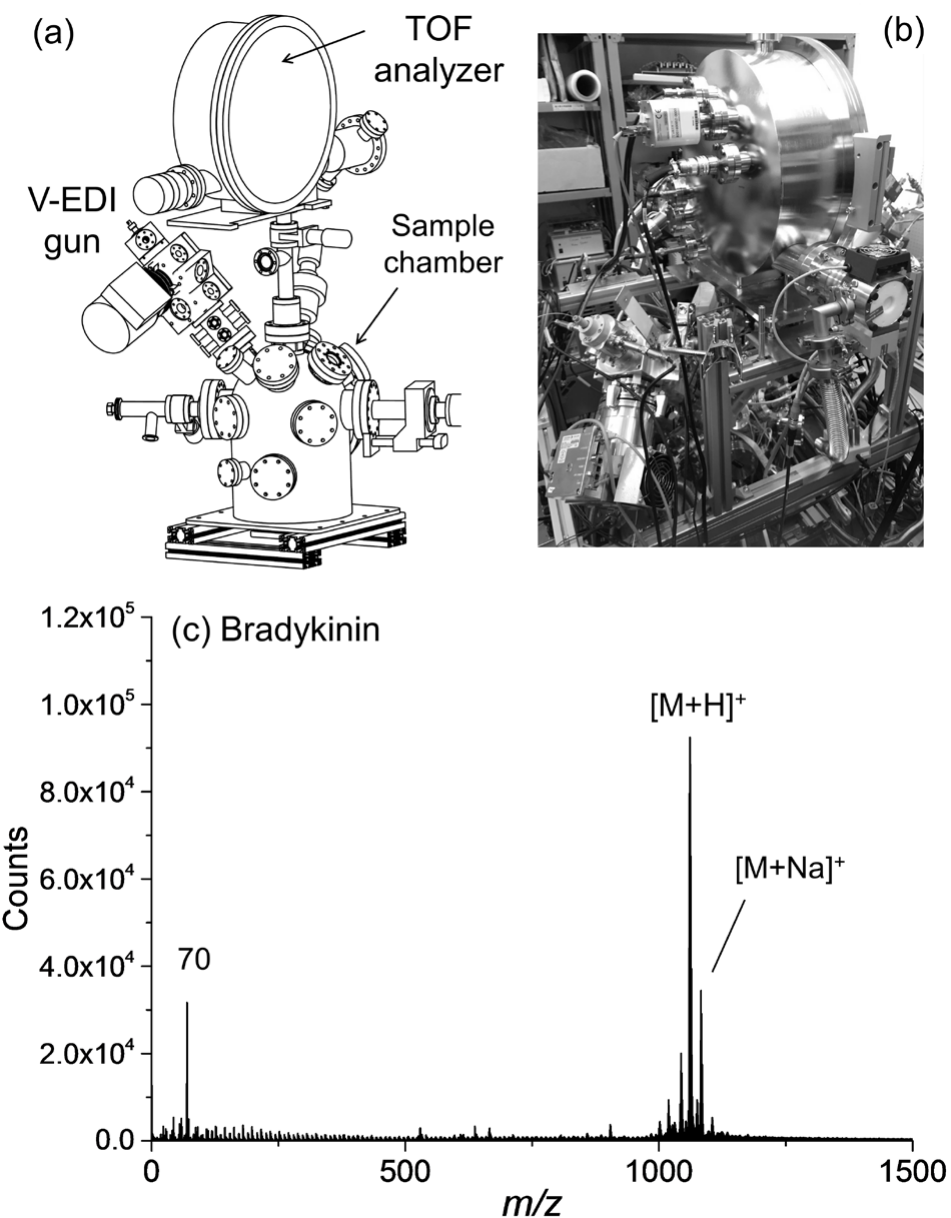
Fig. 6. Schematic diagram (a) and photograph (b) of the experimental setup for SIMS with a triple focusing TOF analyzer. Secondary ion spectrum for the thin bradykinin sample produced by 8 kV V-EDI beam (c). The V-EDI gun was installed in the sample chamber and was connected to the TOF analyzer.

We have begun to use the V-EDI gun as a primary ion beam for TOF-SIMS analysis.^42)^ The secondary ions produced by the V-EDI beams were analyzed using a triple focusing TOF analyzer (TRIFT, ULVAC-PHI, Chigasaki, Japan). A schematic and a photograph of the experimental setup for the TOF-SIMS are shown in Figs. 6(a) and 6(b), respectively. The V-EDI gun was installed in the sample chamber and was connected to the TOF analyzer. The sample chamber and the TOF analyzer were evacuated by a 700 L/s TMP (HiPace700, Pfeiffer) and an 70 L/s TMP (HiPace80, Pfeiffer), respectively, and both were backed up by a 200 L/min RP (RV12, Edwards). The base pressures in the sample chamber and the TOF analyzer were 1×10^−6^ and 8×10^−6^ Pa, respectively, while the working (V-EDI gun ON) pressures were 3×10^−4^ and 1×10^−5^ Pa, respectively. The electrospray emitter used in this study was made of uncoated fused silica (i.d. 15 μm, New Objective). An aqueous solution of pure water with ethanol (water : ethanol=4 : 1) and 0.01 M TFA was supplied through the emitter from a pressure-regulated liquid reservoir (gauge pressure 0.1–0.2 M Pa).^49)^ The lasers were not used in this study because the electrospray of this solution in a vacuum was very stable without laser irradiation. The incident angle and the working distance of the V-EDI gun were 40° to the surface normal and 35 mm, respectively. A 3 mm diameter aperture was installed in the middle part of the V-EDI gun. The other parameters for the V-EDI gun in this study were as follows: accelerating voltage 8.0 kV; electrospray voltage 2.0 kV; condenser lens 1.8 kV; and objective lens 2.3 kV. In this study, secondary ion yield is defined as detected secondary ion counts divided by the number of primary ions (detected ions/primary ion). To calculate the primary V-EDI dose, the average charge state of 287 was adopted because the mean charge state of the V-EDI beam was evaluated from the number of the impact marks observed in scanning electron microscopy and atomic force microscopy.^50)^ The *m*/*z* distributions of the primary V-EDI beams were also measured with a linear TOF analyzer in the previous study. The top of the *m*/*z* distribution was around the peak at 40,000.^50)^ From these results, the typical mass of the droplet ion is calculated to be 1.15×10^7^ u. If the droplet ion is assumed to consist only of water, the chemical formula of the droplet ion is [(H_2_O)_638000_+287H]^287+^. Therefore, the typical total energy and energy per water molecule of the V-EDI were 2.30 MeV and 3.61 eV/H_2_O, respectively.

In general, TOF-SIMS is performed using primary beam pulsing, and the timing of the pulse provides a start signal for the TOF measurement. The pulsed primary beam is incident on the sample, and the emitted secondary ions are intermittently transported through a flight tube and finally detected by the detector. The TOF measurement is completed by using the timing of the detection as the stop signal. Therefore, the time resolution of the TOF-SIMS measurement depends directly on the pulse width of the primary beam. The pulse width on the sample is usually set to be shorter than 10 ns and to have around 10 kHz frequency.^51)^ However, a shortly pulsed (<1 μs) beam could not be obtained for the V-EDI gun, because the droplet ion beams produced by vacuum electrospray have wide *m*/*z* distributions, and the *m*/*z* scales of the droplets were found to be 10^3^–10^6^.^41)^ Even if the droplet ion beams are chopped to a width of less than 10 ns, the pulsed beam width spreads beyond 1 μs before reaching the sample due to the *m*/*z* (velocity) difference of the droplet ions. Therefore, in this study, the timing of the sample-bias pulsing (2.8 kV) to transport the secondary ions into the TOF analyzer was used as a start signal for the TOF-SIMS measurement.^42)^ For the sample-bias pulsing, the secondary ions were accelerated towards the TOF analyzer only in the period when the high voltage was applied. The secondary ions were accelerated by the sample voltage and were post accelerated to a kinetic energy of 8 keV just before being detected with a microchannel plate. Each TOF measurement was taken for 15 s with the TOF-SIMS system. The secondary ion spectra for the Cu sample were measured with the pulsing of the primary beam and the sample bias using conventional Ga ion beams, and similar secondary ion yields were obtained with both pulsing methods.^42)^ Therefore, it was concluded that the TOF-SIMS measurement with sample bias pulsing was suitable for evaluating the secondary ion yields without considering the secondary ion transmission efficiency of the analyzer.

TOF-SIMS spectra were obtained for several biomolecular samples by pulsing the sample bias during continuous irradiation with V-EDI. The secondary ion mass spectrum of the thin bradykinin sample produced by 8 kV V-EDI (current: 2.5 nA, pulse width: 20 ns, frequency: 7.9 kHz) is shown in Fig. 6(c). The bradykinin (C_50_H_73_N_15_O_11_, 1,060.2 u) was purchased from Wako Pure Chemical Industries, Ltd. (Osaka, Japan), and it was used as received. The thin bradykinin film was prepared by casting 10 μL of 1 wt% ethanol solution on a Si wafer and air-dried at room temperature. The mass resolution using the sample bias pulsing method was around 500. With V-EDI, an extremely high intensity of protonated bradykinin ([M+H]^+^) was obtained as shown in Fig. 6(c), and the intensity was much higher than that of the typical fragment *m*/*z* 70. The secondary ion yield of [M+H]^+^ measured with the TOF-SIMS system was calculated to be 1.37. In this study, [M+H]^+^ counts were integrated for two mass units because of the high contribution of the ^13^C isotope. The [M+H]^+^ yield produced by the V-EDI beam was more than two orders of magnitude higher than for Bi_3_^+^ measured with a commercial TOF-SIMS system (nanoTOF, ULVAC-PHI).^42)^ For all the samples measured in this study, the highest secondary ion yields were obtained by the V-EDI beam. From the above results, it is concluded that the sensitivity of SIMS analysis should be dramatically improved by using the V-EDI beam.

## CONCLUSION

In this paper, a brief review on the development of a vacuum electrospray droplet ion (V-EDI) gun for SIMS was given. To improve the performance of the previous A-EDI gun, we have proposed to use vacuum electrospray of aqueous solutions as a beam source. It was demonstrated that vacuum electrospray of the aqueous solutions could be realized by maintaining appropriate vacuum conditions (<0.05 Pa) to prevent the electric discharge and by heating the tip of the capillary with laser to prevent freezing of the aqueous solutions. Based on this technique, we designed the prototype of the V-EDI gun as a novel massive cluster ion gun for SIMS in which the beam source was a vacuum electrospray of an aqueous solution. The beam properties of the V-EDI gun were measured, and the beam current and density were much improved compared to those for the A-EDI gun. The prototype of the V-EDI gun was also connected to a TOF analyzer, and secondary ion spectra produced by the V-EDI beams were measured using a sample-bias pulsing method. The secondary ion yields of biomolecular samples produced by the V-EDI beams were evaluated, and the yields were found to be much higher than those produced with the typical cluster ion beams. However, the V-EDI beam focusing is not enough compared with the typical primary ion beams in SIMS, and further development will be conducted on this issue.

## Notes

Mass Spectrom (Tokyo) 2018; 7(1): A0069

## Notes

Mass Spectrometry Society of Japan bestowed the MSSJ Research Award on Satoshi Ninomiya. This is an invited review of the achievement.
